# Effects of Ti Substitution by Zr on Microstructure and Hydrogen Storage Properties of Laves Phase AB_2_-Type Alloy

**DOI:** 10.3390/ma18153438

**Published:** 2025-07-22

**Authors:** Xiaowei Guo, Lingxing Shi, Chuan Ma, Wentao Zhang, Chaoqun Xia, Tai Yang

**Affiliations:** 1 School of Materials Science and Engineering, Hebei University of Technology, Tianjin 300130, China; 15847951973@163.com (X.G.); slx13616041551@163.com (L.S.); albertma1222@163.com (C.M.); 18866461235@163.com (W.Z.); chaoqunxia@hebut.edu.cn (C.X.); 2 Tianjin Key Laboratory of Laminating Fabrication and Interface Control Technology for Advanced Materials, Hebei University of Technology, Tianjin 300130, China

**Keywords:** hydrogen storage alloy, C14 Laves phase, elemental substitution, kinetics, cycling performance

## Abstract

In order to improve the hydrogen storage properties of Laves phase AB_2_-type alloys, a series of Ti_1−*x*_Zr*_x_*Mn_1.0_Cr_0.85_Fe_0.1_ (*x* = 0.1–0.5) alloys were prepared by arc melting. The effects of Zr content on microstructure and hydrogen storage properties was investigated in detail. Crystal structure characterizations confirmed that all the alloys exhibit a single-phase C14 Laves structure, and the lattice parameters increase with increasing Zr content. The hydrogen storage measurements of the alloys indicate that with increasing Zr content, the hydrogen storage capacity initially increases and then decreases. The hydrogen absorption and desorption measurements of the alloys were performed by a Sieverts-type apparatus. Pressure–composition–temperature (*P-C-T*) tests at various temperatures showed that all the alloys display sloped plateaus. Increasing Zr content results in a gradual decrease in hydrogen absorption and desorption plateau pressures. Moreover, these alloys exhibit varying degrees of hysteresis, which also becomes more pronounced with a rise in Zr content. In summary, the Ti_0.7_Zr_0.3_Mn_1.0_Cr_0.85_Fe_0.1_ alloy demonstrates the best comprehensive hydrogen storage capacity. Further investigation on the cyclic performance of the Ti_0.7_Zr_0.3_Mn_1.0_Cr_0.85_Fe_0.1_ alloy was conducted. It was found that the alloy particles undergo significant pulverization after hydrogenation cycles, but the alloy maintained good phase structure stability and hydrogen storage performance.

## 1. Introduction

Hydrogen energy has the advantages of cleanliness and high efficiency, and holds great application prospects in the context of the growing energy dilemma [1,2]. The storage of hydrogen is crucial for its application. Solid-state hydrogen storage materials exhibit significantly enhanced hydrogen storage capacities and demonstrate efficient hydrogen absorption/desorption cycling under moderate pressure and within mild temperature intervals, making them a safer and more effective alternative to conventional hydrogen storage methods [3,4,5]. Alloys hydrides are a good choice for solid-state hydrogen storage materials, including AB_5_-type, AB-type, AB_2_-type, and A_2_B-type alloys, etc. [6,7,8,9]. Among them, AB_2_-type alloys can be reversibly and safely stored to release hydrogen quickly at room temperature and exhibit excellent thermodynamic and kinetic properties that are of great interest [10,11].

TiMn_2_-based alloys are a typical example of AB_2_-type hydrogen storage alloys [12], and numerous related studies have been conducted. These alloys offer advantages such as simple preparation, decent hydrogen absorption and desorption kinetics, mild operating temperature, and low economic cost. However, issues such as difficulties in activation and high plateau pressure limit their practical applications [13]. The performance of AB_2_-type alloys can be enhanced by optimizing their composition through partial elemental substitution and stoichiometric adjustment [14]. These modifications optimize the plateau pressure by optimizing thermodynamic parameters. Furthermore, hydrogen absorption/desorption kinetics have been enhanced through controlled lattice expansion and improved hydrogen affinity [15,16].

Doping with other elements typically increases the unit cell volume, facilitating the diffusion of hydrogen atoms and thereby changing the hydrogen absorption/desorption plateau pressure of the materials [14]. For example, elements such as Sc, Ti, V, Fe, Cu, Y, Ce, Pr, and Hf can be used to partially replace the A-site elements [17,18,19,20,21,22]. Elements like Al, Si, V, Fe, Co, Ni, Cu, Mo, and W can substitute for B-site elements, enhancing the hydrogen storage performance [14,23,24]. Ni et al. [25] systematically investigated the influence of elements M = Fe, Co, Ni, Cu, and Mo on the hydrogen storage performance of the Ti_0.9_Zr_0.1_Mn_0.95_Cr_0.7_V_0.2_M_0.15_ alloy. By partial substitution of B-site elements, they found that the substitution of the element Ni provided the maximum hydrogen absorption capacity and stable cycling performance, while M = Mo exhibited the quickest hydrogen absorption rate and lowest plateau pressure. Xiu et al. [26] replaced Ti with Y in a Ti_0.6_Zr_0.4_Cr_0.6_Mn_1.4_ alloy and found that the activation characteristics of metal alloys can be optimized through the incorporation of rare earth elements, which also contributes to the enhancement of hydrogen retention capabilities. Compared with the alloy without the addition of Y, the Ti_0.552_Y_0.048_Zr_0.4_Cr_0.6_Mn_1.4_ alloy demonstrates improved cycling performance, achieving a capacity retention of over 96% after 100 hydrogen absorption/desorption cycles. Theoretical analysis and first-principles simulations revealed that Y can make the structural of the system more unstable, which is conducive to hydrogen evolution. In the Ti_40−*x*_V_40+*x*_Fe_15_Mn_5_ (*x* = 0, 2, 4, 6, 8) alloy system, the increased substitution of Ti with V leads to an improvement in the hydrogen storage capacity of the material. However, the reduction in Ti content led to a decrease in the unit cell volume, which made it more difficult for hydrogen atoms to enter unit cell interstices. As a result, the alloy was less responsive to the hydrogen environment, which further leads to slower hydrogen absorption and desorption of the alloy. This indicated that the lattice constant of the hydrogen storage alloys could be optimized to improve thermodynamic properties and significantly enhance hydrogen storage properties [27]. Zeng et al. [28] investigated the effect of substituting Mn with Cr to improve the hydrogen storage performance of TiMn_2_-based alloys. After testing the hydrogen storage properties of Ti_1.25_Mn_1.75−*x*_Cr*_x_* (*x* = 0, 0.05, 0.15, 0.25), the alloy with *x* = 0.15 demonstrated the greatest hydrogen absorption. The partial replacement of Mn with Cr effectively reduced the hysteresis of the alloy without compromising its kinetic performance. The analysis suggested that the introduction of Cr lowered the plastic deformation ability of the alloy, thereby reducing the energy barrier during the hydrogen absorption process.

Changing the properties of materials by varying the stoichiometry of AB_2_-type alloys has also been extensively studied. Different B/A ratios cause the material to change in the production of hydrides, and the chemical environment of the unit cell interstice becomes more complex and changes in the size of the unit cell interstices [29,30,31]. Zhang et al. [32] developed a (Ti_0.85_Zr_0.15_)*_x_*Mn_0.8_CrFe_0.2_ alloy, which was found to display superior hydrogen storage performance under hyper-stoichiometric conditions. Super-stoichiometric ratios have been shown to help reduce the plateau pressures for hydrogen desorption, making alloys more reactive with hydrogen. Additionally, the change in stoichiometric ratio increases the cell volume and reduces hindrance for hydrogen atoms leaving the unit cell interstice, which reduces the hysteresis of the alloy. Zeng et al. [33] investigated the effects of changing the ratio of Ti to Mn elements on reducing alloy costs and improving hydrogen storage performance in the Ti_1+*x*_Mn_2−*x*_ (*x* = 0.20, 0.25, 0.30, 0.35, 0.40, 0.45) alloy. They found that when *x* is in the range of 0.25 to 0.3, a small amount of Ti can partially substitute for Mn sites. However, excessive Ti leads to the precipitation of *α-*TiMn and *β*-Ti phases in the alloy. Nayebossadri et al. [34] investigated the effect of changing stoichiometry of alloys on their hydrogen storage cycling performance and found that the cycling stability of the hyper-stoichiometric alloy Ti_30.6_V_16.4_Mn_48.7_(Zr_0.7_Cr_0.8_Fe_2.8_) was higher than that of stoichiometric and hypo-stoichiometric alloys. They also observed that changing the alloy from hyper-stoichiometric to hypo-stoichiometric resulted in the production of stabilized hydride phases and enhanced heterogeneous lattice strain. Chen et al. [35] synthesized a series of V_35_Ti*_x_*Mn_65−*x*_ (*x* = 20, 25, 30, 35, and 40) hydrogen storage alloys, in which increasing Ti/Mn ratio led to an increase in the cell volume and a decrease in the hydrogen desorption pressure of the alloys. Expansion in unit cell volume facilitates the easier insertion of hydrogen atoms into a unit cell interstice, which accelerates the rate of hydrogen absorption and makes the hydrides it forms more stable. Manickam et al. [36] studied the alloys (Ti_0.65_Zr_0.35_)_1+*x*_MnCr_0.8_Fe_0.2_ (*x* = 0, 0.05, 0.075, and 0.1) and found that the alloy (Ti_0.65_Zr_0.35_)_1.1_MnCr_0.8_Fe_0.2_ has the highest hydrogen storage capacity. The maximum hydrogen absorption capacity of the alloy has a proportional relationship with the value of the A/B ratio of elements, while the plateau pressure and plateau slope of the *P-C-T* curve decrease as the A/B ratio of elements decreases. This indicates that the increase in alloy stoichiometry alters the chemical environment at the unit cell interstice, thereby modifying the thermodynamic properties of the material.

In summary, proper elemental substitution and stoichiometric adjustments have a positive impact on providing more unit cell interstices for hydrogen atoms and improving the hydrogen absorption and desorption kinetics rate of the alloy to enhance their hydrogen storage performance. Based on TiMn_2_-type alloys, this study adopts a partial substitution strategy to design Ti_1−*x*_Zr*_x_*Mn_1.0_Cr_0.85_Fe_0.1_ alloys (*x* = 0.1, 0.2, 0.3, 0.4, 0.5). By varying the elemental ratios of different alloys, the differences in their crystal structures and phase compositions were compared. Additionally, the hydrogen absorption and desorption kinetics and thermodynamic properties of the five alloys were systematically tested under different temperature conditions, focusing on the analysis of hydrogen absorption/desorption plateau pressure, plateau slope rate, and hysteresis. Further testing was conducted on the alloy with the most optimal hydrogen storage performance for its cycling stability, and its phase changes were examined.

## 2. Materials and Methods

### 2.1. Sample Preparation

Raw materials with a purity of 99%, including titanium, zirconium, manganese, chromium, and iron, were melted in a vacuum arc furnace according to their respective proportions. The vacuum arc melting furnace (model WK-II) was manufactured by Physcience Opto-electronics Co., Ltd., Beijing, China. The total weight for each alloy was about 20 g. To ensure uniform alloy composition, titanium, zirconium, chromium, and iron, with similar melting points, were first melted together, followed by mixing with manganese for further melting and flipping. Each sample was melted and flipped at least five times to ensure homogeneity. The melting current was set to 180 A, and the base pressure of the chamber was below 4.5 × 10^−3^ Pa before backfilling with argon. Finally, five alloy ingots with the nominal chemical composition of Ti_1−*x*_Zr*_x_*Mn_1.0_Cr_0.85_Fe_0.1_ alloys (*x* = 0.1, 0.2, 0.3, 0.4, 0.5) were prepared. For simplicity, the alloys are referred to as Zr01, Zr02, Zr03, Zr04, and Zr05 samples.

### 2.2. Structural Characterizations

Powder X-ray diffraction (XRD) analysis, using a Bruker D8 Discover diffractometer from Bruker Corporation (Karlsruhe, Germany) with Cu-K_α_ radiation, was employed to determine the phase composition of the alloys. The morphology of alloy ingots and powders was investigated using a JSM-7100F scanning electron microscope (JEOL Ltd., Tokyo, Japan) equipped with an energy-dispersive spectrometer (EDS) system. To enable the better backscattered electron (BSE) characterization of the alloy cross-sections, the samples were cut into 2 mm thick slices using arc cutting. The slices were sequentially ground with sandpapers of increasing grit sizes from 1000 to 5000, followed by polishing with a polishing solution.

### 2.3. Hydrogen Absorption and Desorption Performance Tests

Before testing the hydrogen absorption and desorption kinetic performance of alloys, the alloy ingots were mechanically ground into powder and passed through a 200-mesh sieve. Hydrogen storage testing of the alloys was conducted at different temperatures using a laboratory-made Sieverts-type apparatus. Each sample was heated to 380 °C under 4 MPa hydrogen pressure for 1 h, then it was cooled to room temperature. Then, the samples were hydrogenated and dehydrogenated three times at room temperature to fully activate the alloy. Isothermal hydrogen absorption and desorption kinetics were tested at 10 °C, 25 °C, and 40 °C. The hydrogen pressure for the hydrogenation kinetic tests was 3 MPa, and the final hydrogen pressure during dehydrogenation tests was about 0.02 MPa. During hydrogen absorption, the instrument’s pressure reading accuracy was 0.001 MPa, while during hydrogen desorption, the pressure gauge’s reading accuracy was 0.0001 MPa. Although the pressure in the chamber began to change at the very beginning of the experiment, the Sieverts-type apparatus started recording data from the sixth second after the test began. Therefore, the hydrogen absorption/desorption curves started at 6 s. After that, the data were recorded in real time without any further delay. *P-C-T* testing involved gradually increasing the hydrogen pressure from 0 MPa to 4 MPa, with pressure values recorded at each stabilized step until the material reached hydrogen absorption saturation. Subsequently, the hydrogen was gradually released until the pressure started to decrease, with pressure values recorded during each stabilization step of the depressurization process, ultimately yielding a curve that shows the relationship between pressure and hydrogen capacity at a constant temperature.

## 3. Results and Discussion

### 3.1. Microstructure Characteristics

**Figure 1:**
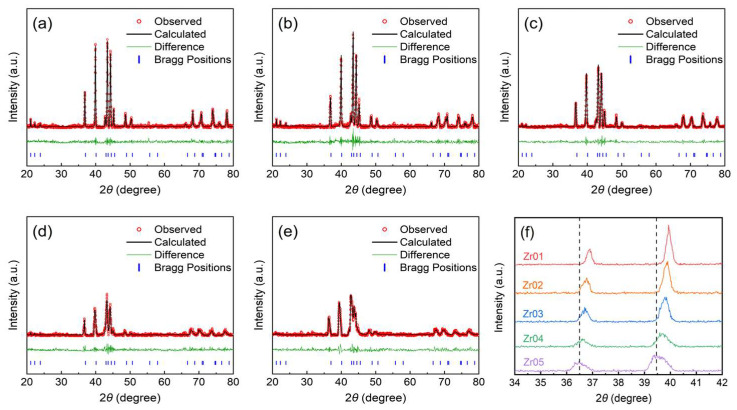
XRD analysis of the alloys: (**a**) Zr01; (**b**) Zr02; (**c**) Zr03; (**d**) Zr04; (**e**) Zr05; (**f**) magnified view.

The XRD patterns of the alloys are shown in Figure 1. All the alloys exhibit a single C14 phase without any obvious diffraction peaks of secondary phases. Moreover, the comparison of the diffraction peak angles of the alloys reveals that the diffraction peaks shift to smaller angles and their intensities decrease accordingly with the increase in Zr content, as shown in Figure 1f. This indicates that increasing Zr content can increase the lattice parameters of the alloys. The XRD data for the alloys were analyzed, and the calculated lattice parameters and unit cell volumes are listed in Table 1. The XRD patterns were fitted using the Rietveld refinement method, assuming a C14 Laves phase structure. The lattice parameters were obtained from the refined structural model, and the unit cell volumes were calculated accordingly [37]. A gradual increase in lattice parameters and unit cell volume was observed with increasing Zr content. With increasing Zr content, a leftward shift in the XRD peaks was observed, as shown in Figure 1f. This shift reflects the above-mentioned trend and can be attributed to the larger atomic radius of Zr compared to that of Ti [19]. The diffraction peak intensity of the alloys decreases with increasing Zr content. This may be attributed to the atomic size mismatch introduced by Zr, which leads to atomic disorder, generating numerous point defects and dislocations. As a result, the lattice distortion is enhanced and the structural order is reduced, thereby lowering the peak intensity [38,39,40].

**Figure 2:**
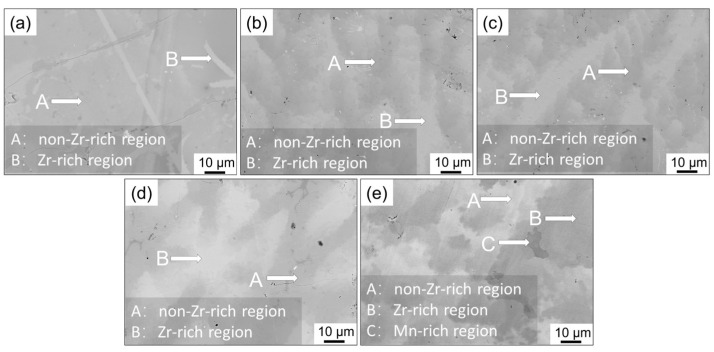
Backscattered electron images of alloy ingots: (**a**) Zr01; (**b**) Zr02; (**c**) Zr03; (**d**) Zr04; (**e**) Zr05.

Figure 2 shows backscattered electron images of the alloy cross-sections with different Zr contents. The results reveal that all the alloys consist of gray-black A regions and gray-white B regions, while the Zr05 alloy additionally contains darker C regions. The brighter and darker regions correspond to areas with different average atomic numbers, indicating phase distribution and elemental segregation within the alloy. Combined with backscattered electron image characteristics, it is confirmed that the A and B regions are associated with variations in Zr content. The Zr content in region B is significantly higher than that in region A. The B regions in the Zr01 alloy are significantly smaller than those in the other alloys. In contrast, the B regions in the other alloys are dispersed throughout the A regions. Table 2 summarizes the elemental composition of the alloy ingot cross-sections obtained by EDS analysis. With increasing Zr content, the proportion of Zr in both regions A and B increased accordingly, but the increase was more pronounced in region B. For the Zr05 alloy, the C regions are associated with the enrichment of Mn. The Mn-rich region C observed in the Zr05 alloy was probably caused by local elemental segregation during solidification. As the Zr content increases, the solidification process becomes more complex and Mn tends to accumulate in certain regions due to its lower melting point. Regions with higher Zr content led to local changes in phase composition and microstructure, resulting in non-uniform hydrogen diffusion pathways. In addition, as the Zr content in the alloy increases, Zr-rich regions become more numerous, and this inhomogeneity becomes more evident.

### 3.2. Hydrogen Absorption and Desorption Kinetic Properties

**Figure 3:**
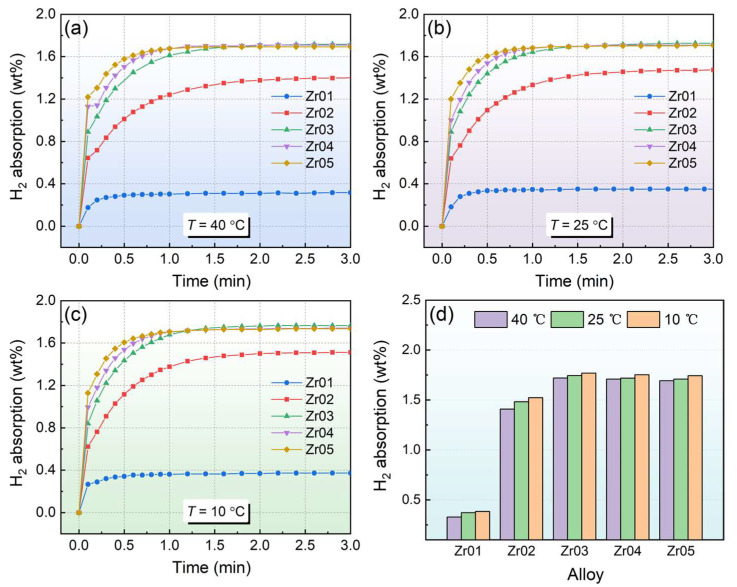
Hydrogen absorption curves of the alloys at different temperatures (**a**) 40 °C; (**b**) 25 °C; (**c**) 10 °C; (**d**) hydrogen absorption capacity of different alloys.

Figure 3 presents the hydrogen absorption kinetics curves for the alloys under different temperature conditions. All the alloys completed hydrogen saturation within 3 min, demonstrating favorable hydrogen absorption kinetics. The hydrogen storage capacity of the alloys initially increases and subsequently decreases with the increase in Zr content. The Zr03 alloy exhibited the highest hydrogen absorption capacity, reaching 1.77 wt% at 10 °C. In contrast, the Zr01 alloy showed poor hydrogen storage performance, absorbing only 0.38 wt% H_2_ at 10 °C, which may be related to the relatively smaller B region observed in its SEM image. However, a further increase in Zr content leads to a decrease in the hydrogen absorption capacity of the alloys. This is mainly because the high atomic mass of Zr weakens the ability to store the hydrogen of the alloys. The Zr03 alloy exhibited the highest hydrogen storage capacity, which can be attributed to the increased Zr content leading to an enlarged unit cell volume and providing larger interstitial sites for hydrogen accommodation [41]. Additionally, Zr atoms have lower electronegativity, making them more likely to lose electrons and form hydrides with hydrogen atoms [17].

**Figure 4:**
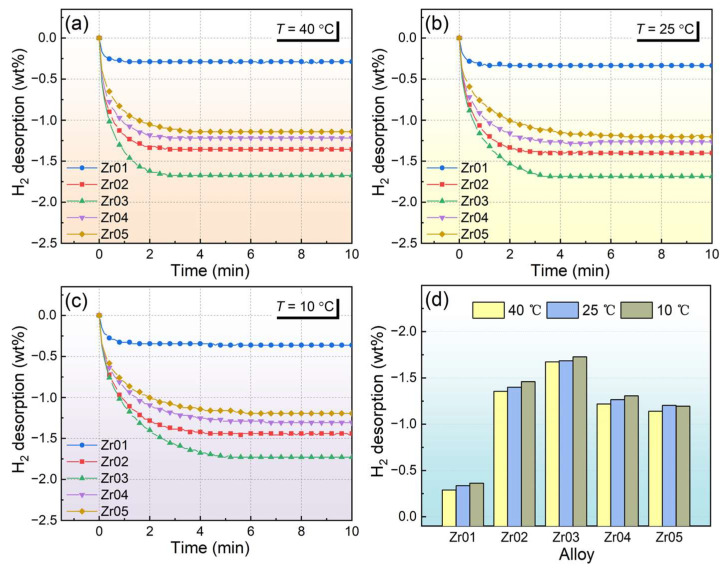
Hydrogen desorption curves of the alloys at different temperatures (**a**) 40 °C; (**b**) 25 °C; (**c**) 10 °C; (**d**) hydrogen desorption capacity of different alloys.

Figure 4 exhibits the hydrogen desorption kinetic curves for the alloys at 40 °C, 25 °C, and 10 °C. All the alloys exhibit good hydrogen desorption rates. The hydrogen desorption rate of the alloys is faster at higher temperatures. At 10 °C, the alloys complete hydrogen desorption within 6 min, while at 40 °C, the time is reduced to around 3 min. This is because hydrogen atoms have lower energy and diffuse more slowly in the alloys at low temperatures. The maximum desorption hydrogen content first increases and then decreases with increasing Zr content. The Zr03 alloy exhibits the highest hydrogen desorption capacity. Figure 4d shows the hydrogen desorption capacities of different alloys at various temperatures. With the further increase in Zr content, the hydrogen desorption capacity of the Zr04 and Zr05 alloys decreases significantly. This phenomenon was also observed in the study by Guo et al. [42]. This may be due to the increase in the mass of the alloys caused by the addition of Zr. In addition, lower dehydrogenation pressure caused by increasing Zr content reduces the hydrogen desorption capacities of the Zr04 and Zr05 alloys. That is, the hydrogenated Zr04 and Zr05 alloys cannot be completely dehydrogenated under the hydrogen desorption testing pressures.

**Figure 5:**
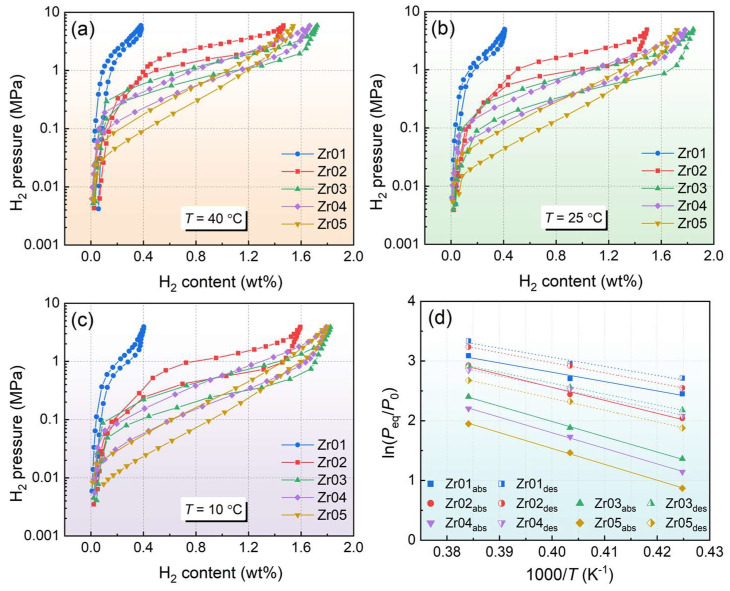
*P-C-T* curves of alloys at different temperatures: (**a**) 40 °C; (**b**) 25 °C; (**c**) 10 °C; (**d**) van ‘t Hoff fitting curves.

The *P-C-T* curves of the alloys were tested at 40 °C, 25 °C, and 10 °C, respectively, and there are obvious sloped plateaus and hysteresis in the *P-C-T* curves of the alloys. As shown in Figure 5, there is an inversely proportional relationship between plateau pressure and Zr content. The Zr01 alloy exhibits the highest plateau pressure as well as the lowest capacity for hydrogen storage. The Zr05 alloy has the minimum hydrogen absorption and desorption plateau pressures under all temperature conditions. The reason is that Zr has a lower electronegativity and an increase in the lattice unit cell interstice, which enhances the ability of alloys to bind with hydrogen atoms [43,44]. This results in alloys with higher Zr content exhibiting lower plateau pressures. The pressure of the sloped hydrogen absorption/desorption plateau was evaluated using the method proposed by Yu et al. [45]. There are two distinct inflection points before and after the inclined plateau pressure, noted as *P*_1_ and *P*_2_. The average of the pressures corresponding to the two points is taken, and it is defined as *P*_eq_.

To further investigate the hydrogen absorption and desorption thermodynamic properties of the alloys, the reaction enthalpy changes (∆*H*) and entropy changes (∆*S*) were calculated using van ‘t Hoff fitting. The equation is as follows [46]:

(1) $$\ln\frac{P_{\mathrm{eq}}}{P_{0}}=\frac{\Delta H}{RT}-\frac{\Delta S}{R}$$

In this equation, *T* is the test temperature, *P*_eq_ is the equilibrium pressure corresponding to the plateau pressure region, *P*_0_ is the standard atmospheric pressure value of 0.10132 MPa, and *R* is the ideal gas constant. By plotting the scatter plot of ln(*P*_eq_/*P*_0_) versus 1000/*T* and performing linear regression analysis, the ∆*H* and ∆*S* of the material can be calculated. Figure 5d shows the van ‘t Hoff fitting curves of ln(*P*_eq_/*P*) vs. 1000/*T* for the absorption and desorption process of the alloys. Table 3 shows the hydrogen absorption and desorption thermodynamic parameter of the alloys. It is found that the enthalpy and entropy changes in the alloys show gradually increasing trends as the Zr content increases. The increase in hydrogenation enthalpy change indicates that more heat is required during the reaction, leading to a higher stability of the alloy hydrides. This makes hydrogen absorption easier, while hydrogen desorption becomes more difficult. The increase in the ∆*H* and ∆*S* is directly related to the shift in the *P-C-T* curves plateau pressure and the increase in unit cell volume caused by the higher Zr content [23,24].

**Figure 6:**
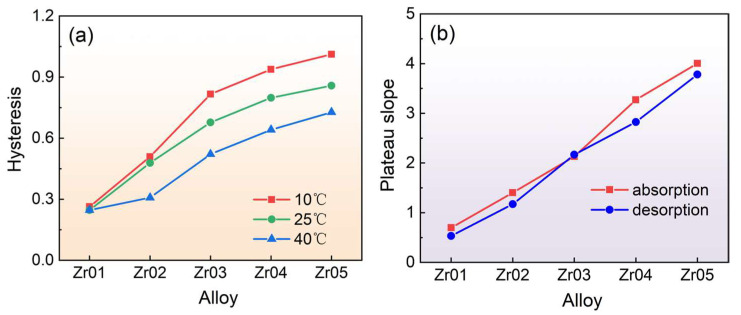
Hysteresis (**a**) and plateau slopes (**b**) for each alloy at 25 °C.

Figure 6 presents the trends of the hysteresis factor (*H*_f_) and plateau slope factor (*S*_f_) at 25 °C as functions of Zr content for the alloys. The hysteresis factor is defined as ln(*P*_a_/*P*_d_). The plateau slope factor is defined as ln(*P*_2_/*P*_1_) using the method referenced by Yu et al. [45]. It can be seen from Figure 6a that the hysteresis factor *H*_f_ increases with the increase in Zr content and the highest hysteresis factor *H*_f_ is found for the Zr05 alloy. According to the study by Fang et al. [47], hysteresis is directly influenced by factors such as alloy reaction temperature, particle size, number of cycles, and annealing treatment. In addition, electronegativity, atomic size, and the electron concentration of the alloy indirectly regulate the hysteresis factor by affecting the Δ*H* of the hydrogen absorption/desorption process. As mentioned earlier, with the increase in Zr content, both the electronegativity and atomic size of the alloy increase, leading to a higher Δ*H* and a larger difference in free energy, which ultimately results in an increase in hysteresis. Moreover, it can be observed in Figure 6b that the plateau slope of the alloys increases with the increase in Zr content. According to the theory proposed by Park et al. [48], the plateau slope factor in alloys is jointly affected by macroscopic segregation and the effects of chemical energy and strain energy at the microscopic level. Macroscopically, this mainly refers to the homogeneity of the alloy composition [49]. Chemical energy is influenced by the ratio of the A and B elements in AB_2_-type alloys and the Zr content, and increasing Zr content often leads to an increase in plateau slope. A higher Zr content also increases the lattice distortion in the alloy, making the unit cell interstice more complex and thereby enhancing the strain energy of the alloy. Both the chemical energy and strain energy have an impact on the plateau slope of the material.

### 3.3. Cycling Performance

**Figure 7:**
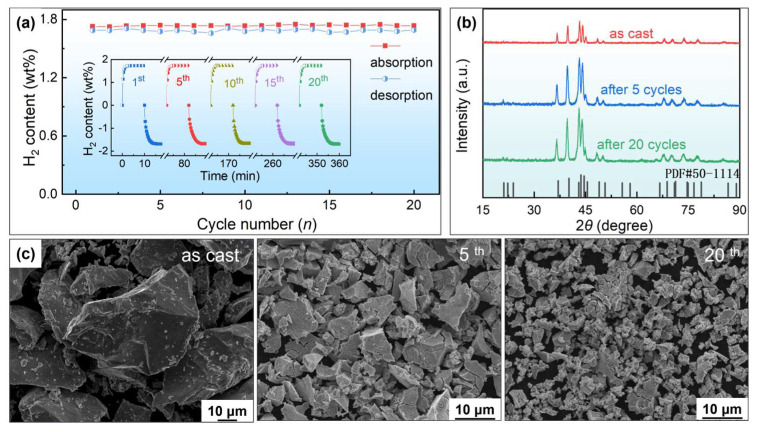
Hydrogen absorption and desorption cycling tests of the Zr03 alloy: (**a**) kinetic curves and reversible hydrogen storage capacities during the first 20 cycles at 25 °C; XRD patterns (**b**) and SEM images (**c**) of the alloy before and after 5 and 20 hydrogenation cycles.

According to the above results, the Zr03 alloy was identified to deliver the most favorable hydrogen storage performance. Consequently, further hydrogenation cycling tests were conducted to observe its cycling stability performance. Figure 7a shows the hydrogen absorption and desorption capacities and kinetics curves of the Zr03 alloy during the first 20 cycles. It was found that the hydrogen storage capacity of the alloy showed almost no degradation, and the hydrogen absorption/desorption rate also showed no significant changes. The slight fluctuations in the hydrogen desorption capacities may be attributed to negligible errors caused by temperature variations and operational factors during the testing process. The XRD and SEM analysis were employed to investigate the phase composition and microstructural changes in the Zr03 alloy at different cycling stages. As shown in Figure 7b, there are no significant changes in the XRD peaks after one or multiple hydrogen desorption cycles, indicating no significant phase transformation or decomposition. These results clearly indicate that the material maintains good stability during repeated hydrogen absorption and desorption cycles. Figure 7c shows a significant reduction in particle size of the alloy after 20 hydrogenation cycles. This can be attributed to the repeated volumetric changes in the alloy particles during the cycling process, leading to breakage of the alloy particles [49]. However, the particle pulverization has no impact on the phase composition of the alloy; therefore, the alloy maintains good cycling stability performance.

## 4. Conclusions

1. A series of TiMn_2_-based alloys with varying Zr contents were prepared, with all these alloys exhibiting a single C14-type Laves phase structure. As the Zr content increased, both the lattice parameters and unit cell volume increased accordingly. SEM and EDS analysis indicates that the Zr distributions in the alloys are not uniform.
2. Kinetic tests showed that the alloys exhibited good hydrogen absorption and desorption rates. The hydrogen storage capacity of the alloy first increases and then decreases with increasing Zr content. The Zr03 alloy demonstrated relatively high hydrogen absorption and desorption capacities, with a maximum hydrogen absorption of 1.77 wt% at 10 °C.
3. Increasing the Zr content has been shown to effectively lower the plateau pressure in the alloys. The van ‘t Hoff fitting demonstrated that the Zr content influences the thermodynamic properties of the alloys, leading to increases in both the enthalpy change and entropy change. Specifically, the Zr05 alloy exhibited the largest absolute values for hydrogen absorption and desorption enthalpy and entropy changes, with values of 19.56 kJ·mol^−1^ H_2_/101.9 J·K^−1^·mol^−1^ H_2_ for absorption, and 26.56 kJ·mol^−1^ H_2_/121.6 J·K^−1^·mol^−1^ H_2_ for desorption. Moreover, the *P-C-T* curves of the alloys exhibit an obvious slope and a certain degree of hysteresis.
4. Significant alloy particle pulverization occurs during the hydrogen absorption and desorption cyclic process. There is no noticeable capacity degradation and phase composition changes during hydrogenation cycles; the results suggest that the Zr03 alloy possesses good cycling stability.

## Figures and Tables

**Table 1:** Lattice parameters and unit cell volumes of the alloys.

Alloys	Lattice Parameters		Unit Cell Volume (Å^3^)
	*a* (Å)	*c* (Å)	
Zr01	4.873	7.999	164.5
Zr02	4.883	8.020	165.6
Zr03	4.899	8.040	167.1
Zr04	4.908	8.406	167.8
Zr05	4.935	8.105	170.9

**Table 2:** Chemical compositions derived from EDS analysis in each alloy.

Alloys	Regions	Element Fraction (at %)					Percentage of Zr Elements	Error %
		Ti	Zr	Mn	Cr	Fe		
Zr01	A	21.50	1.57	17.81	25.31	1.79	2.31%	2.81%
	B	26.79	2.20	23.01	32.86	2.05	2.53%	2.74%
Zr02	A	29.95	3.56	34.80	29.05	2.64	3.56%	2.80%
	B	26.99	5.29	32.07	32.91	2.74	5.29%	2.80%
Zr03	A	22.75	4.60	31.89	26.01	1.81	5.28%	3.12%
	B	18.29	7.23	28.61	28.46	2.43	8.50%	2.54%
Zr04	A	18.55	4.06	42.12	21.06	1.54	5.65%	2.68%
	B	14.06	10.14	35.28	24.92	1.73	11.77%	2.14%
Zr05	A	26.44	7.50	36.99	26.28	2.78	7.50%	3.20%
	B	13.97	15.02	33.10	34.89	3.02	15.02%	2.64%
	C	4.60	0.01	54.50	28.77	0.78	0.01%	2.70%

**Table 3:** The hydrogen absorption and desorption thermodynamic properties of the alloys.

Alloys	∆*H*_abs_ (kJ·mol^−1^ H_2_)	∆*S*_abs_ (J·K^−1^·mol^−1^ H_2_)	∆*H*_des_ (kJ·mol^−1^ H_2_)	∆*S*_des_ (J·K^−1^·mol^−1^ H_2_)
Zr01	−15.23	−91.5	15.64	90.7
Zr02	−16.85	−97.1	21.73	112.5
Zr03	−18.31	−99.5	25.54	122.0
Zr04	−18.77	−100.7	26.05	122.2
Zr05	−19.56	−101.9	26.56	121.6

## Data Availability

The original contributions presented in the study are included in the article. Further inquiries can be directed to the corresponding author.
